# Physicians as Experts and Counsel

**Published:** 1889-03

**Authors:** 


### PHYSICIANS AS EXPERTS AND COUNSEL.

Judge Willard Bartlett, of the New York Supreme Court, recently read a very interesting paper on "Some Relations of Scientific Experts to the Administration of the Law," before the Medico-Legal Society of New York. One thing that Judge Bartlett specially emphasized was that physicians should not act as experts and counsel at the same time. "The wise doctor, as it seems to me, will take care not to act in both capacities. If he is to testify in the case he will not act as assistant counsel; if he acts as assistant counsel he will keep off the witness stand. There is no good reason why the most distinguished physician should not place his professional knowledge at the service of one of the parties to a litigation which involves questions of medical science. To do so cannot justly subject him to reproach, but it does lessen his fitness and his usefulness as a witness in that litigation. In assisting counsel he will inevitably come to share the sentiments of counsel as to the result. Just as counsel will seek to bring out every fact that may prove beneficial to the cause of his client, and will endeavor to destroy, as far as may be, the effect of any proof which is injurious to him, so the medical man thus employed will suggest questions for the examination of witnesses on his own side, and for the cross-examination of witnesses on the other side, which will call out answers favorable to the party at whose instance he has been brought into the case.

All this goes on before the jury, who fully comprehend where the medical questions really come from. If afterward they see the doctor take the witness stand it is impossible that they should regard him as otherwise than prejudiced. However truthful his testimony may be, and however correct his opinions, his evidence is the evidence of a partisan, and this fact invariably detracts from its force and effect. When, therefore, a litigant or his lawyer applies to a physician or surgeon for assistance as an expert, the medical man should first ascertain whether it is desired that he shall be a witness in the case or shall act as adviser to counsel. If he be asked to appear as a witness he should carefully abstain from seeing the injured person or examining into the circumstances upon any agreement or understanding that his right to compensation for so doing is to depend upon the conclusion he reaches. I have reason to belive that cases are not unknown in which the counsel for the plaintiff has said to the physician: 'I think my client's injuries are serious and that he will never be a well man again. Go and see him for me. If you find he is not permanently hurt I shall not expect you to charge me anything for making the examination; but if you conclude that his injuries are incurable I will call you as a witness on the trial and pay you handsomely.' "

Of course no honorable physician will accept such a proposition as this; he will insist that his compensation shall not be dependent upon the opinion he may form. His services are worth just so much, whatever his opinion may be. The physician that conforms to the honorable course is always prepared for the most severe cross-examination; and if in the cross-examination the fact is brought out that the party by whom he was called agreed to pay him for examining the injured person, whatever might be the opinion formed by the examination, this evidence will be of great weight with the jury. The attitude of the medical man called as an expert should be as nearly impartial as possible. His sole desire should be to be true in his statements of fact, and sound in his expressions of opinion.

"It is well known that many railroad companies," says Judge Bartlett, "employ medical men continuously under salary to investigate all cases of personal injury which occur upon their lines, and to assist in the trial of the lawsuits to which such casualties give rise. The testimony of these witnesses is much less weighty and effective with a jury than the evidence of physicians called in for the particular case only, and I have more than once heard counsel ask: 'How long would the doctor continue to be employed by the railroad company unless he always testified in its favor?' Physicians thus permanently employed to treat accident cases are also peculiarly exposed to damaging cross-examination. But I have no disposition to criticise railroad companies or other corporations upon whose property accidents are liable to occur for employing physicians to protect them against false or exaggerated claims on account of personal injuries. Indeed, they are also instrumental in procuring the adjustment of well-founded claims, and no doubt hundreds of suits are settled every year upon the recommendation of the medical advisers of railway corporations. Instances sometimes occur, however, of gross invasions of private right by physicians so employed, who have been known to force their way into the houses and to the very bedsides of injured persons, without regard to the protests of their families, in order to ascertain the extent of the injuries immediately after they were inflicted, and to prepare themselves to testify on the subject if a suit should subsequently be brought. These trespasses are committed only against people in the humbler walks of life, who hardly know that they can rightfully expel the intruder under such circumstances. They are wholly without justification or excuse, and it is satisfactory to find that juries pay but little attention to medical testimony founded upon these unwelcome visits. In his well-known History of the Criminal Law of England, Sir James Fitz-James Stephen, one of the most distinguished judges of the High Court of Justice of the present time, suggests a course of conduct on the part of the medical witnesses in accident cases which he thinks would do away with the reproach which attaches to expert testimony because the witnesses are so often merely counsel in disguise. 'If medical men,' he says, 'laid down for themselves a positive rule that they would not give evidence unless before doing so they met in consultation the medical men to be called on the other side, and exchanged their views fully, so that the medical witnesses on the one side might know what was to be said by the medical witnesses on the other, they would be able to give a full and impartial account of the case which would not provoke cross-examination.' He truly observes that such a practice implies a high standard of honor and professional knowledge on the part of the witnesses called to give evidence, and the suggestion would seem almost Utopian, were it not that the writer adds: 'For many years this course has been invariably pursued by all the most eminent physicians and surgeons in Leeds, and the result is that in trials at Leeds (where actions for injuries in railway accidents and the like are very common) the medical witnesses are hardly ever cross-examined at all, and it is by no means uncommon for them to be called on one side only.' This is the best possible answer to the objection that the plan assumes a higher standard of conduct than is practicable. Experience has shown that it does not; and what English medical men can bring about in the borough of Leeds, American medical men ought surely to be able to accomplish."

Judge Bartlett then spoke of the laws in regard to the commitment and confinement of the insane. A physician is not justified, he says, "in certifying to the insanity of a person he is called to see, simply because some member of the patient's family gives him a long history of the hallucinations of the invalid, or because some other doctor assures him that the man is mad. He must examine into the case himself to the best of his ability and in the light of all the medical knowledge he possesses. If he does this he will incur no liability even for an erroneous judgment. If he does less than this, and certifies that a person is insane who turns out to have been sane at the time, he can be held responsible by the injured party. Too much care can hardly be taken by physicians in the preparation of lunacy certificates, or by judges in passing upon them when presented for approval. In my opinion the judge should always require the attendance of one of the physicians before him, if not both, and should personally inquire as to the facts in addition to the matters set out in the cerficate. I am aware that some physicians deem it a hardship to be compelled to attend court for this purpose, but such should let others act in their stead. The approval of the certificate by the judge was not intended to be only a formality. In fact, no act in the administration of this portion of the lunacy laws can properly be deemed a mere matter of form. The popular distrust of the management of lunatic asylums, however, has always been so great, and the horrors attending the incarceration of a sane person in a mad house appeal so vividly to the imagination, that the State could well afford to do something to assure the public from time to time that none but lunatics are prisoners in these institutions. This might be accomplished through the supervision of the courts, to whose care the interests of all persons of unsound mind are expressly committed by statute. If in each of the five judicial departments into which the State is divided the Supreme Court were empowered to appoint a qualified expert in insanity every year, whose duty it should be personally to examine every person imprisoned as a lunatic within that department and report the results of his examination to the court, it would be rendered tolerably certain that no abuses were being perpetrated, and the assurance would be worth all it might cost in the way of compensation to the five physicians for their services."

